# Supplementary material for: Resolving the full spectrum of human genome variation using Linked-Reads
Source: Genome Res. 2019 Apr;29(4):635–45. doi: 10.1101/gr.234443.118 (PMC6442396; doi:10.1101/gr.234443.118)
Supplement: Supplemental Material [file supp_gr.234443.118_Supplemental_longranger-2.2.zip › longranger-2.2.tar.bz2!/lr2.2-public/lib/stan/cmdstan/stan/doc/api/html/rev_2functions_2atan2_8hpp_source.html]

Stan: src/stan/agrad/rev/functions/atan2.hpp Source File


|  |  |
| --- | --- |
|  | Stan  2.5.0  probability, sampling & optimization |


- Main Page
- Related Pages
- Namespaces
- Classes
- Files

- File List
- File Members

All Classes Namespaces Files Functions Variables Typedefs Enumerations Enumerator Friends Macros Pages

- src
- stan
- agrad
- rev
- functions

atan2.hpp

Go to the documentation of this file.

1 #ifndef STAN\_\_AGRAD\_\_REV\_\_FUNCTIONS\_\_ATAN2\_HPP

2 #define STAN\_\_AGRAD\_\_REV\_\_FUNCTIONS\_\_ATAN2\_HPP

3

4 #include <valarray>

5 #include <stan/agrad/rev/var.hpp>

6 #include <stan/agrad/rev/internal/vv\_vari.hpp>

7 #include <stan/agrad/rev/internal/vd\_vari.hpp>

8 #include <stan/agrad/rev/internal/dv\_vari.hpp>

9 #include <math.h>

10

11 namespace stan {

12  namespace agrad {

13

14  namespace {

15  class atan2\_vv\_vari : public op\_vv\_vari {

16  public:

17  atan2\_vv\_vari(vari\* avi, vari\* bvi) :

18  op\_vv\_vari(::atan2(avi->val\_,bvi->val\_),avi,bvi) {

19  }

20  void chain() {

21  double a\_sq\_plus\_b\_sq = (avi\_->val\_ \* avi\_->val\_) + (bvi\_->val\_ \* bvi\_->val\_);

22  avi\_->adj\_ += adj\_ \* bvi\_->val\_ / a\_sq\_plus\_b\_sq;

23  bvi\_->adj\_ -= adj\_ \* avi\_->val\_ / a\_sq\_plus\_b\_sq;

24  }

25  };

26

27  class atan2\_vd\_vari : public op\_vd\_vari {

28  public:

29  atan2\_vd\_vari(vari\* avi, double b) :

30  op\_vd\_vari(::atan2(avi->val\_,b),avi,b) {

31  }

32  void chain() {

33  double a\_sq\_plus\_b\_sq = (avi\_->val\_ \* avi\_->val\_) + (bd\_ \* bd\_);

34  avi\_->adj\_ += adj\_ \* bd\_ / a\_sq\_plus\_b\_sq;

35  }

36  };

37

38  class atan2\_dv\_vari : public op\_dv\_vari {

39  public:

40  atan2\_dv\_vari(double a, vari\* bvi) :

41  op\_dv\_vari(::atan2(a,bvi->val\_),a,bvi) {

42  }

43  void chain() {

44  double a\_sq\_plus\_b\_sq = (ad\_ \* ad\_) + (bvi\_->val\_ \* bvi\_->val\_);

45  bvi\_->adj\_ -= adj\_ \* ad\_ / a\_sq\_plus\_b\_sq;

46  }

47  };

48  }

49

64  inline var atan2(const var& a, const var& b) {

65  return var(new atan2\_vv\_vari(a.vi\_,b.vi\_));

66  }

67

80  inline var atan2(const var& a, const double b) {

81  return var(new atan2\_vd\_vari(a.vi\_,b));

82  }

83

121  inline var atan2(const double a, const var& b) {

122  return var(new atan2\_dv\_vari(a,b.vi\_));

123  }

124

125  }

126 }

127 #endif

dv\_vari.hpp

vd\_vari.hpp

stan::agrad::var::vi\_

vari \* vi\_

Pointer to the implementation of this variable.

**Definition:** var.hpp:40

var.hpp

stan::agrad::atan2

fvar< T > atan2(const fvar< T > &x1, const fvar< T > &x2)

**Definition:** atan2.hpp:16

stan::agrad::var

Independent (input) and dependent (output) variables for gradients.

**Definition:** var.hpp:27

vv\_vari.hpp


---

[ Stan Home Page ]

*© 2011–2014,
Stan Development Team.*
